# Identification of Three Genes Associated with Metastasis in Melanoma and Construction of a Predictive Model: A Multiracial Identification

**DOI:** 10.1155/2022/4567063

**Published:** 2022-05-21

**Authors:** Ying Chen, Dan Wang, Qingyun Li, Yiyi Zhang, Zheng Peng, Yu He, Bin Lin, Meifang Xu, Qiong Chen, Yang Chen

**Affiliations:** ^1^Department of Plastic Surgery, Dermatology Hospital of Fuzhou, Fuzhou, China; ^2^Department of Radiology, Taikang Tongji (Wuhan) Hospital, Wuhan, China; ^3^Department of Colorectal Surgery, Fujian Medical University Union Hospital, Fuzhou, China; ^4^Department of Colorectal Surgery, The First Affiliated Hospital of Fujian Medical University, Fuzhou, China; ^5^Department of Pathology, Fujian Medical University Union Hospital, Fuzhou, China; ^6^Department of Pharmacy, The Affiliated Hospital of Putian University, Putian, China

## Abstract

The aim of this study was to identify hub genes associated with metastasis and prognosis in melanoma. Weighted gene coexpression network analysis (WGCNA) was performed to screen and identify hub genes. ROC and K-M analyses were used to verify the hub genes in the internal and external data sets. The risk score model and nomogram model were constructed based on the IHC result. Through WGCNA, the three hub genes, SNRPD2, SNRPD3, and EIF4A3, were identified. In the external data set, the hub genes identified were associated with the worse prognosis (TCGA, SNRPD2, *P* ≤ 0.02; SNRPD3, *P* = 0.12; EIF4A3, *P* = 0.11; GSE65904, SNRPD2, *P* = 0.04; SNRPD3, *P* = 0.10; EIF4A3, *P* < 0.01; GSE19234, SNRPD2, *P* < 0.01; SNRPD3, *P* < 0.01; EIF4A3, *P* < 0.01). In the GSE8401, we found that the hub genes were highly expressed in the metastasis compared with the nonmetastasis group (SNRPD2, 988.5 ± 47.83 vs. 738.4 ± 35.35, *P* < 0.01; SNRPD3, 502.7 ± 25.7 vs. 416.4 ± 23.88, *P* = 0.02; EIF4A3, 567.6 ± 19.56 vs. 495.2 ± 21.1, *P* = 0.01). Moreover, the hub genes were identified by the IHC in our data set. The result was similar with the external data set. The hub genes could predict the metastasis and prognosis in the Chinese MM patients. Finally, the GSEA and Pearson analysis demonstrated that the SNRPD2 was associated with the immunotherapy. The three hub genes were identified and validated in MM patients in external and internal data sets. The risk factor model was constructed and verified as a powerful model to predict metastasis and prognosis in MM patients.

## 1. Introduction

Melanoma (MM) is a highly aggressive malignant tumor that is prone to metastasis at an early stage and has the highest mortality rate among skin cancers; it leads to more than 90% of skin cancer-related deaths [1, 2]. The American Cancer Society reports that about 106,110 new cases are diagnosed in 2021 in the US [3]. Nowadays, the surgery combined with the immunotherapy, radiotherapy, and chemotherapy had been increased the prognosis of the MM patients [4]. However, the prognosis of the MM with metastasis patients was still poor [5]. Recently, several studies have paid attention on metastatic MM, and many potential biomarkers have been reported to predict the metastasis of the MM [6–8]. However, the majority of the biomarkers were according to the skin MM, and the mucous MM has been ignored. Thus, to identify the biomarkers which could predict the metastasis of the skin MM and mucous MM is needed.

In Europe and the US, most of the MM occurred in the skin. But it was different in China; the location of MM often occurred in other mucous tissues other than the skin, including the head and neck (nasal pharyngeal and oral), gastrointestinal (upper GI and lower GI), gynecological, and urological [9]. To search the effective biomarkers which could predict the metastasis not only in skin MM but also in the mucous MM was important. Currently, the weighted gene coexpression network analysis (WGCNA) is a widely used algorithm, and it was unbiased systematic biological approach which can be used to screen the effective biomarker in the multiple diseases including various cancers. Moreover, it could bridge the gap between individual genes and clinical information [10–12]. Thus, the WGCNA could be used as an efficient algorithm to screen the hub genes for the metastatic MM.

In this context, this study was aimed at screening the relevant hub genes for metastatic MM, including skin MM and mucous MM, by using WGCNA. Then, the hub genes were verified using patient tissue samples and internal and external testing data sets.

## 2. Materials and Methods

### 2.1. Data Collection from the GEO and TCGA Databases

**Figure 1:**
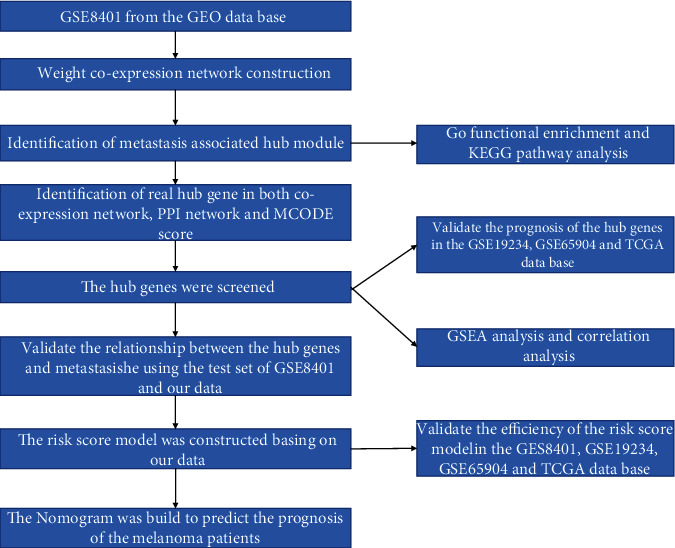
Work flow diagram in this study. GO: Gene Ontology; GSEA: gene set enrichment analysis; PPI: protein-protein interaction; WGCNA: weighted gene coexpression network analysis.

Three microarray data sets were downloaded from the Gene Expression Omnibus (GEO) database (https://www.ncbi.nlm.nih.gov/geo/). The data set (GSE8401) [13, 14] from Austria included mRNA expression profiles of 31 primary MM samples and 52 metastatic MM samples treated as a training set for WGCNA. Data set GSE19234 [15] from the USA included mRNA expression profiles of 44 MM patients, and GSE65904 [16, 17] from Sweden included mRNA expression profiles of 210 MM patients which were used to verify the hub gene prognosis in MM. In addition, The Cancer Genome Atlas (TCGA) data set from the US contained with 462 MM patients was also used to verify the prognosis of the hub genes. A flow diagram of the present study is demonstrated in Figure 1.

### 2.2. Data Collection and Immunohistochemical Analysis

A total of 147 MM patients between January 2010 and December 2018 were enrolled in our study. The patients were diagnosed with MM by the two individual pathologists. The protocol was approved by the Committee of the Fujian Medical University Union Hospital.

The protein expression of hub genes in 147 MM patients was assessed using the immunohistochemical streptavidin-biotin complex method [18]. The IHC score was described in our previous study [18, 19]. The score between 0 and 4 was defined as the low expression and >4 was defined as high expression. All analyses were performed in a double-blind manner.

### 2.3. Coexpression Network Construction

The WGCNA algorithm was described in detail previously [12]. Briefly, firstly the coexpression network was constructed by “WGCNA” package in R software [20, 21]. Next, the correlation matrix was established and the soft threshold power was determined. Then, the topological overlap matrix (TOM) was established [22–24]. Based on the metastasis or not, we calculated each module *P* value by the *t*-test gene significance. The module which was most associated with the metastasis was selected as the hub module. The green module was selected.

### 2.4. Hub Gene Identification

The hub genes should be considered as the maximum specific weight and core genes of the interaction in the green module of all genes. To identify hub genes, we uploaded all genes in the green module to the Search Tool for the Retrieval of Interacting Genes (STRING) to construct protein-protein interaction (PPI) network. Cytoscape was used to perform PPI network analysis to screen hub gene in the maximum specific weigh modules within the PPI network. The Molecular Complex Detection (MCODE) analysis was performed to screen the hub genes. Based on the PPI, Pearson analysis, and MCODE analysis, we screened three hub genes, SNRPD2, SNRPD3, and EIF4A3.

### 2.5. GSEA GO Enrichment and KEGG Pathway Analysis

To explore the potential function of hub genes in MM patients, GSEA was performed in MM patients from GSE8401 data sets. *P* < 0.05 and |enrichment score (ES) | > 0.3 were set as the cutoff criteria.

The Gene Ontology (GO) analysis and Kyoto Encyclopedia of Genes and Genomes (KEGG) pathway analysis were performed using standard enrichment computation method.

### 2.6. Statistical Analysis

Statistical analysis was performed using the SPSS software (24 SPSS), GraphPad Prism 7, and R software (4.0.3). Continuous variables were reported in means and standard deviation via the analysis of variance test. Survival outcomes were assessed using the Kaplan-Meier method and log-rank test. The optimal cutoff values for hub gene expression were determined using the X-tile program (http://www.tissuearray.org/rimmlab/). The TIMER database was used to analyze the relationship between the hub genes and tumor-infiltrating immune cells [25, 26]. The risk factor model was constructed based on the Cox proportional hazard model [27]. *P* < 0.05 was considered statistically significant.

## 3. Results

### 3.1. Construction of Weighted Coexpression Network and Identification of Key Modules

**Figure 2:**
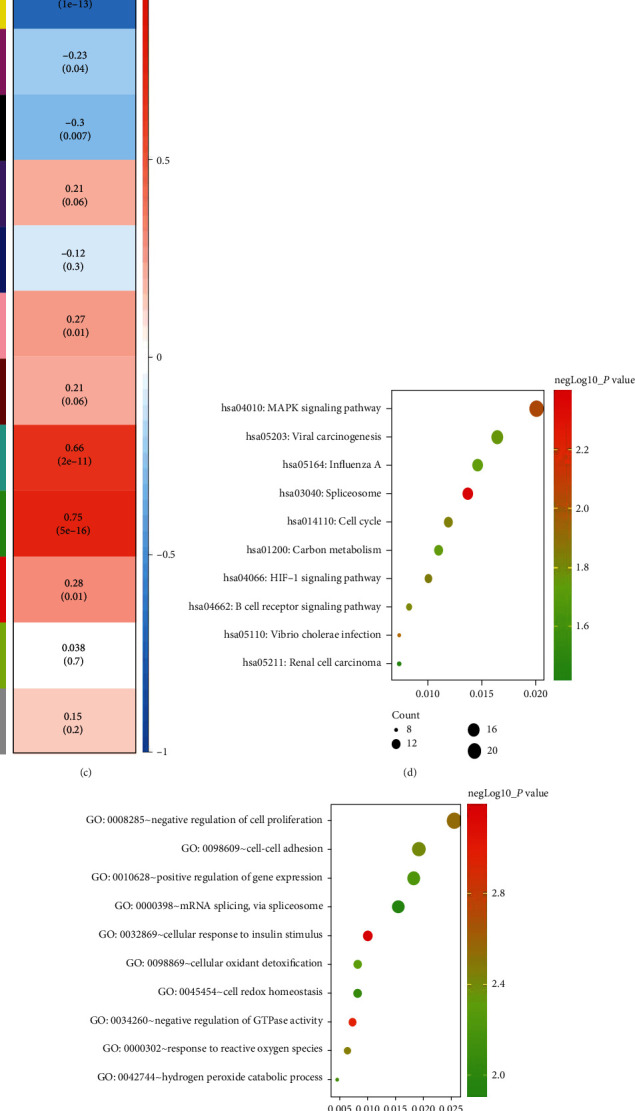
Weighted gene coexpression network analysis (WGCNA) and hub gene screened. (a) Dendrogram of genes based on a dissimilarity measure. (b) The scatter diagram of the relationship between the hub green module and metastasis. (c) Heatmap of the correlation between all modules and metastasis. (d) KEGG pathway analysis of genes in green modules. (e) GO functional analysis of genes in green modules. (f) Protein-protein interaction network of genes which had the highest score in the PPI degree in the green module.

To identify the hub gene, we used the weighted coexpression network to analyze the GSE8401 (Figure 2(a)). A total of 12 modules were identified (Figure 2(c)), and the green module has the highest positive association with the metastasis (Figure 2(b)) (*r* = 0.83, *P* < 0.01). Thus, the green module was selected as the hub modules for further analysis. KEGG analysis was conducted to investigate the pathway of the green module. Genes in the green module were mainly enriched for MAPK signaling pathway, cell cycle signaling pathway, and spliceosome signaling pathway (Figure 2(d)). GO analysis was conducted to investigate the pathway of the green module. Genes in the green module were mainly enriched for mRNA splicing, cell proliferation, and cell-cell adhesion (Figure 2(e)).

### 3.2. Hub Gene Identification and Validation in the GSE8401 Data Set

There were 715 genes in the green module considered as candidate genes. Firstly, the genes were analyzed by the PPI network and 214 genes were considered as the candidate hub genes, based on the PPI degree score and MCODE score (all selected the top10 genes) (Figure 2(f)). Finally, we chose the most associated genes, SNRPD2, SNRPD3, and EIF4A3, as the “real” hub genes.

**Figure 3:**
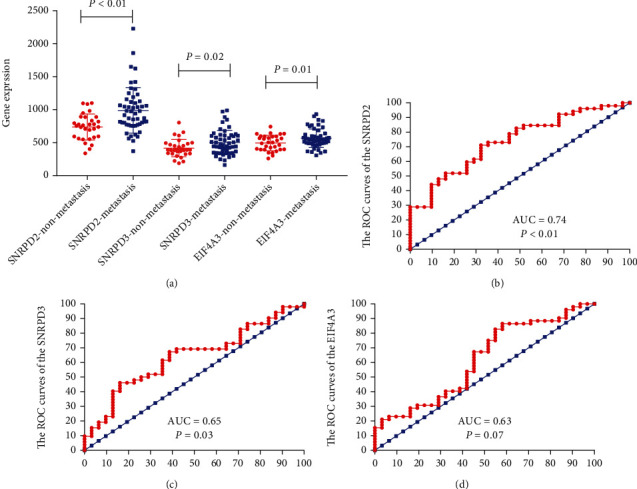
Validation of the hub genes in the GSE8401. (a) The hub gene expression in the metastasis and nonmetastasis group in the GSE8401 (metastasis group vs. nonmetastasis group (SNRPD2, 988.5 ± 47.83 vs. 738.4 ± 35.35, *P* < 0.01; SNRPD3, 502.7 ± 25.7 vs. 416.4 ± 23.88, *P* = 0.02; EIF4A3, 567.6 ± 19.56 vs. 495.2 ± 21.1, *P* = 0.01). (b–d) ROC curves and AUC statistics to evaluate the predictive efficiency of the hub genes in GSE8401 to distinguish metastasis from nonmetastasis in melanoma patients: (b) SNRPD2, AUC = 0.74, *P* < 0.01; (c) SNRPD3, AUC = 0.65, *P* = 0.03; and (d) EIF4A3, AUC = 0.63, *P* = 0.03.

To validate the hub genes, we examined SNRPD2, SNRPD3, and EIF4A3 expressions in the GSE8401 data set between metastasis MM and nonmetastasis MM. The result demonstrated that the SNRPD2, SNRPD3, and EIF4A3 were all highly expressed in the metastasis group compared with the nonmetastasis group (SNRPD2, 988.5 ± 47.83 vs. 738.4 ± 35.35, *P* < 0.01; SNRPD3, 502.7 ± 25.7 vs. 416.4 ± 23.88, *P* = 0.02; EIF4A3, 567.6 ± 19.56 vs. 495.2 ± 21.1, *P* = 0.01; Figure 3(a)). Moreover, the ROC curve also demonstrated that SNRPD2, SNRPD3, and EIF4A3 could efficiently predict the metastasis in MM patients (SNRPD2, *P* < 0.01, AUC = 0.74; SNRPD3, *P* = 0.03, AUC = 0.65; EIF4A3, *P* = 0.07, AUC = 0.63; Figures 3(b)–3(d)).

### 3.3. Hub Gene Validation in the External Data Sets

**Figure 4:**
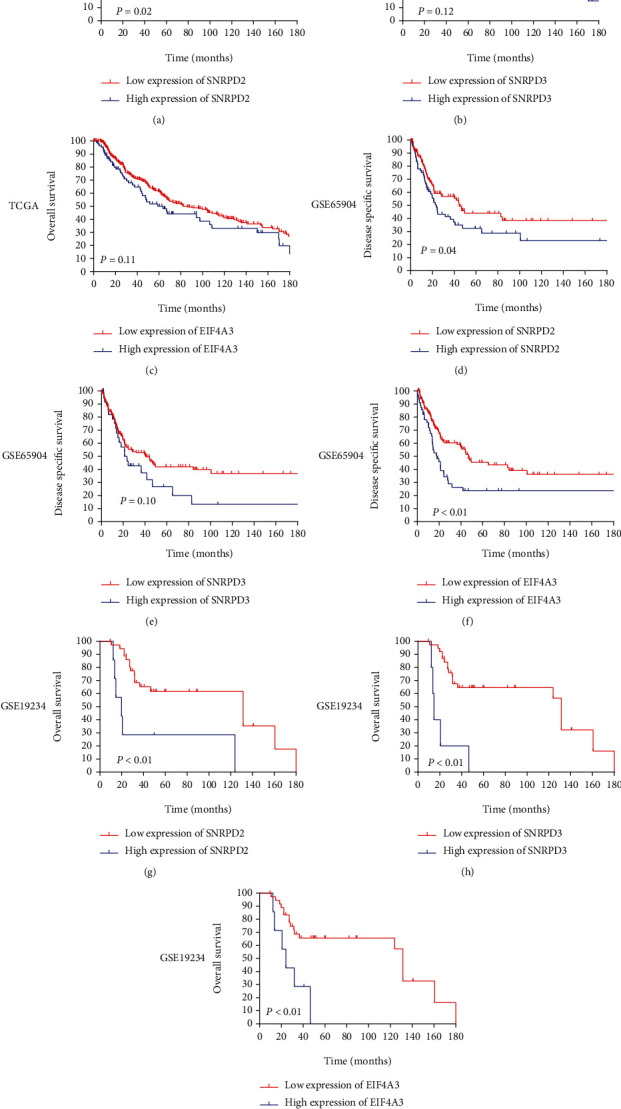
Low hub gene expression was correlated with a better prognosis in the external data sets. (a–c) Low hub gene expression was correlated with a better prognosis in the TCGA data sets: (a) SNRPD2, *P* = 0.02; (b) SNRPD3, *P* = 0.12; and (c) EIF4A3, *P* = 0.11. (d–f) Low hub gene expression was correlated with a better prognosis in the GSE65904 data sets: (d) SNRPD2, *P* = 0.04; (e) SNRPD3, *P* = 0.10; and (f) EIF4A3, *P* < 0.01. (g–i) Low hub gene expression was correlated with a better prognosis in the GSE19234 data sets: (g) SNRPD2, *P* < 0.01; (h) SNRPD3, *P* < 0.01; and (i) EIF4A3, *P* < 0.01.

To further explore the prognostic impact of SNRPD2, SNRPD3, and EIF4A3 on the MM patients, the X-tile program was used to determine the cutoff value of SNRPD2, SNRPD3, and EIF4A3. In the TCGA data set, the X-tile result identified that the best cutoffs of the hub genes were as follows: SNRPD2, 6.492; SNRPD3, 5.490; and EIF4A3, 4.805, respectively. The result demonstrated that the higher expression of the SNRPD2 had worse OS compared with the lower group (SNRPD2, *P* ≤ 0.02Figure 4(a)), but there is no significance in the SNRPD3 and EIF4A3 (SNRPD3, *P* = 0.12; EIF4A3, *P* = 0.11; Figures 4(b) and 4(c)). Similarly, in the GSE65904, the X-tile result identified that the best cutoffs of the hub genes were as follows: SNRPD2, 7500; SNRPD3, 2450; and EIF4A3, 2950, respectively. The result demonstrated that the higher expression of the SNRPD2 and EIF4A3 had worse OS compared with the lower group (SNRPD2, *P* = 0.04; EIF4A3, *P* < 0.01; Figures 4(d) and 4(f)), but there is no significance in the SNRPD3 (SNRPD3, *P* = 0.10; Figure 4(e)). In the GSE19234, the X-tile result identified that the best cutoffs of the hub genes were as follows: SNRPD2, 14099.5; SNRPD3, 509.2; and EIF4A3, 3334.0, respectively. The result demonstrated that the higher expression of the hub genes had worse OS compared with the lower group (SNRPD2, *P* < 0.01; SNRPD3, *P* < 0.01; EIF4A3, *P* < 0.01; Figures 4(g)–4(i)).

### 3.4. Hub Gene Validation in Our Data Set by the Immunohistochemical

**Figure 5:**
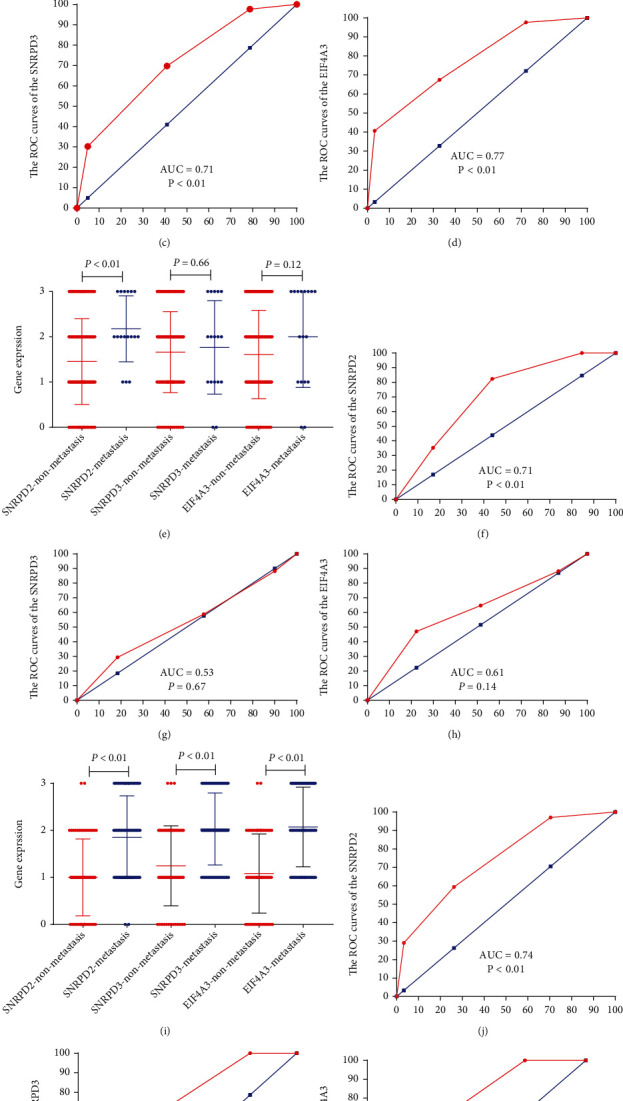
Validation of the hub genes in our data set by IHC. (a) The hub gene expression in the nonmetastasis melanoma patients and metastasis melanoma in our data set (all *P* < 0.01). (b–d) ROC curves and AUC statistics to evaluate the predictive efficiency of the hub genes to distinguish diseases free from metastasis in melanoma patients: (b) SNRPD2, AUC = 0.76, *P* < 0.01; (c) SNRPD3, AUC = 0.71, *P* < 0.01; and (d) EIF4A3, AUC = 0.77, *P* < 0.01. (e) The hub gene expression in the pathology stage IV melanoma patients and pathology stage I-III melanoma in our data set (SNRPD2, *P* < 0.01; SNRPD3, *P* = 0.66; EIF4A3, *P* = 0.12). (f–h) ROC curves and AUC statistics to evaluate the predictive efficiency of the hub genes to distinguish pathology stage IV from pathology stage I-III in melanoma patients: (f) SNRPD2, AUC = 0.71, *P* < 0.01; (g) SNRPD3, AUC = 0.53, *P* = 0.67; and (h) EIF4A3, AUC = 0.61, *P* = 0.14. (i) The hub gene expression in the nonrecurrence melanoma patients and recurrence melanoma in our data set (all *P* < 0.01). (j–l) ROC curves and AUC statistics to evaluate the predictive efficiency of the hub genes to distinguish diseases free from metastasis in melanoma patients: (j) SNRPD2, AUC = 0.74, *P* < 0.01; (k) SNRPD3, AUC = 0.73, *P* < 0.01; and (l) EIF4A3, AUC = 0.77, *P* < 0.01.

In the previous study, we screened and identified the hub genes in the US and Europe. However, the data from Asia was still lacking. To further independently validate the hub genes in Asia, we examined the expression level of SNRPD2, SNRPD3, and EIF4A3 in the MM patients from China. The IHC analysis demonstrated that the hub gene proteins were highly expressed in the metastasis group compared with the disease-free group (*P* < 0.01) (Figure 5(a)). The grade of the SNRPD2, -, +, ++, and +++, in the disease-free group was 18, 27, 14, and 2, respectively, and the grade -, +, ++, and +++ in the metastasis group was 2, 29, 29, and 26, respectively. The grade of the SNRPD3, -, +, ++, and +++, in the disease-free group was 13, 22, 22, and 3, respectively, and the grade -, +, ++, and +++ in the metastasis group was 2, 24, 36, and 26, respectively. The grade of the EIF4A3, -, +, ++, and +++, in the disease-free group was 17, 24, 18, and 2, respectively, and the grade -, +, ++, and +++ in the metastasis group was 2, 26, 23, and 35, respectively. Moreover, the ROC curve also demonstrated that SNRPD2, SNRPD3, and EIF4A3 could efficiently predict the metastasis in MM patients (SNRPD2, *P* < 0.01, AUC = 0.76; SNRPD3, *P* < 0.01, AUC = 0.71; EIF4A3, *P* < 0.01, AUC = 0.77; Figures 5(b)–5(d)).

Moreover, the IHC analysis also demonstrated that the SNRPD2 protein was highly expressed in the concurrent metastases group compared with the concurrent nonmetastasis group (*P* < 0.01) (Figure 5(e)). The SNRPD3 and EIF4A3 proteins have no difference between the metastasis group and nonmetastasis group (SNRPD3, *P* = 0.66; EIF4A3, *P* = 0.12) (Figure 5(e)). The grade of the SNRPD2, -, +, ++, and +++, in the nonmetastasis group was 20, 53, 35, 22, respectively, and the grade -, +, ++, and +++ in the metastasis group was 0, 3, 8, and 6, respectively. The grade of the SNRPD3, -, +, ++, and +++, in the nonmetastasis group was 13, 42, 51, and 24, respectively, and the grade -, +, ++, and +++ in the metastasis group was 2, 5, 5, and 5, respectively. The grade of the EIF4A3, -, +, ++, and +++, in the nonmetastasis group was 17, 16, 38, and 29, respectively, and the grade -, +, ++, and +++ in the metastasis group was 2, 4, 3, and 8, respectively. The ROC curve also demonstrated that SNRPD2 could efficiently predict the metastasis in MM patients (SNRPD2, *P* < 0.01, AUC = 0.71, Figure 5(f)). However, SNRPD3 and EIF4A3 proteins could not predict the metastasis in MM patients (SNRPD3, *P* = 0.67, AUC = 0.53, Figure 5(g); EIF4A3, *P* = 0.14, AUC = 0.61; Figure 5(h)).

The IHC analysis demonstrated that the hub gene proteins were highly expressed in the recurrence group compared with the nonrecurrence group (*P* < 0.01) (Figure 5(i)). The grade of the SNRPD2, -, +, ++, and +++, in the nonrecurrence group was 18, 27, 14, and 2, respectively, and the grade -, +, ++, and +++ in the recurrence group was 2, 26, 21, and 20, respectively. The grade of the SNRPD3, -, +, ++, and +++, in the nonrecurrence group was 13, 22, 22, and 3, respectively, and the grade -, +, ++, and +++ in the recurrence group was 0, 19, 29, and 21, respectively. The grade of the EIF4A3, -, +, ++, and +++, in the nonrecurrence group was 17, 24, 18, and 2, respectively, and the grade -, +, ++, and +++ in the recurrence group was 0, 22, 20, and 27, respectively. Moreover, the ROC curve also demonstrated that SNRPD2, SNRPD3, and EIF4A3 could efficiently predict the recurrence in MM patients (SNRPD2, *P* < 0.01, AUC = 0.74; SNRPD3, *P* < 0.01, AUC = 0.73; EIF4A3, *P* < 0.01, AUC = 0.77; Figures 5(j)–5(l)).

An analysis of the relationship between the three hub genes and prognosis in MM patients further revealed that the high expression of SNRPD2, SNRPD3, and EIF4A3 was associated with worse OS (all *P* < 0.01, Figures 5(a)–5(c)). We found similar result in the DFS analysis; the lower expression of hub genes had better prognosis compared with the higher group (all *P* < 0.01, Figures 5(d)–5(f)).

### 3.5. Construction of a Risk Factor Model and Validation

**Figure 6:**
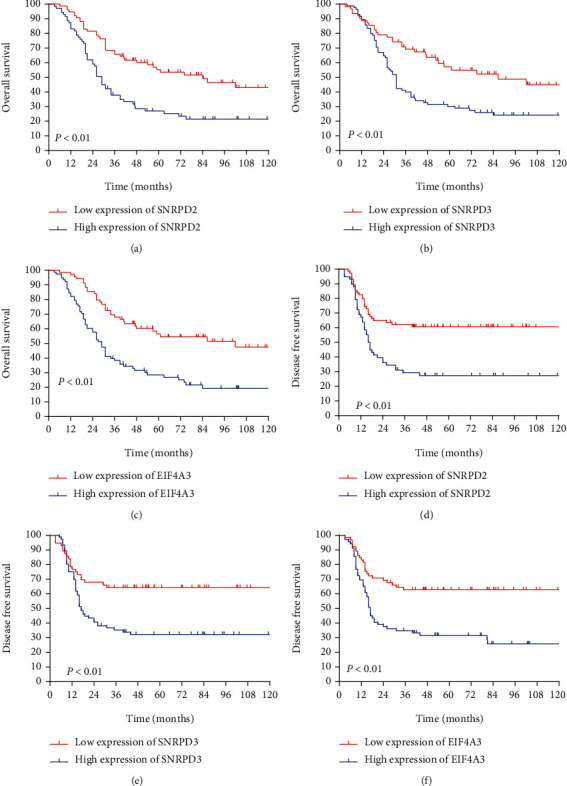
Low hub gene expression was correlated with a better prognosis in all data set. (a–c) Low hub gene expression was correlated with a better overall survival (all *P* < 0.01). (d–f) Low hub gene expression was correlated with a better disease-free survival (all *P* < 0.01).

**Figure 7:**
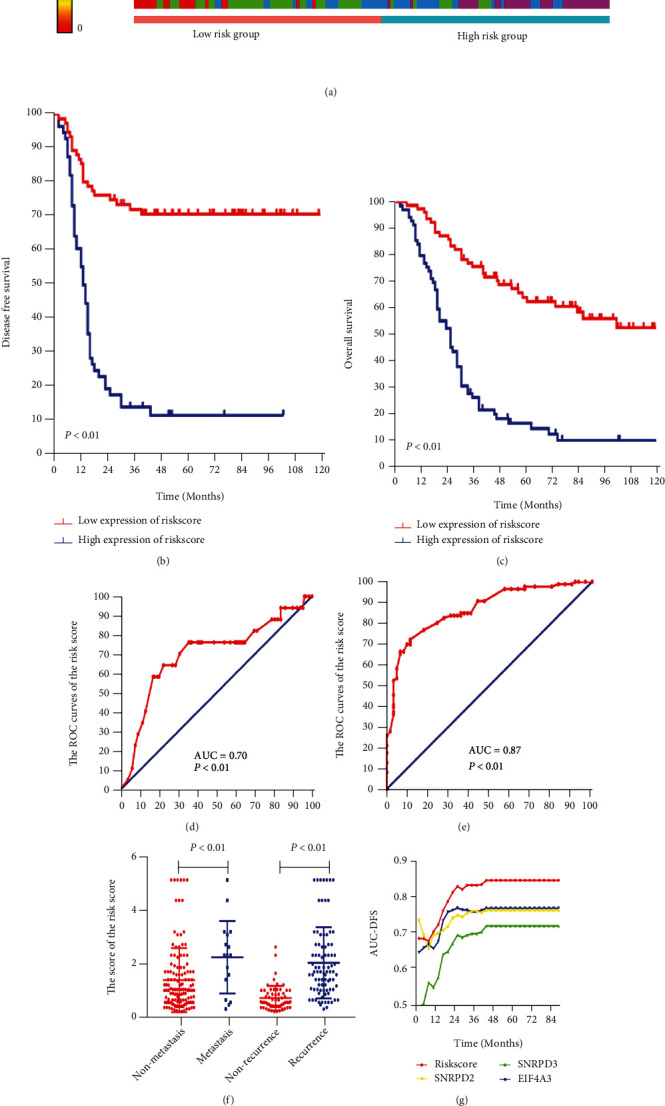
Risk factor model construction verified in our data set. (a) The risk factor model of the hub genes in the 147 melanoma patients. Upper: risk score distribution of 147 melanoma patients. Middle: status of every patient in our data set (*N* = 147). Lower: expression heatmap of the hub genes corresponding to each sample above. (b) The DFS analysis of the risk score in the 147 melanoma patients (*P* < 0.01). (c) The OS analysis of the risk score in the 147 melanoma patients (*P* < 0.01). (d and e) ROC curves and AUC statistics to evaluate the predictive efficiency of the risk score model: (d) distinguishing pathology stage IV from pathology stage I-III in melanoma patients (AUC = 0.70, *P* < 0.01) and (e) distinguishing diseases free from metastasis in melanoma patients (AUC = 0.87, *P* < 0.01). (f) The risk score in the melanoma patients (metastasis group vs. nonmetastasis group: 2.172 ± 0.327 vs. 1.322 ± 0.1047, *P* < 0.01; recurrence group vs. disease-free group: 1.966 ± 0.143 vs. 0.6503 ± 0.05832, *P* < 0.01). (g and h) Time-dependent AUC curves of the hub genes and risk factor models for the prediction of DFS (g) and OS (h).

A Cox regression analysis performed to explore the disease-free survival impact of the hub genes in MM patients demonstrated that the SNRPD2 (HR = 1.603, 95% CI: 1.236-2.079, *P* < 0.001), SNRPD3 (HR = 1.174, 95% CI: 0.904-1.525, *P* = 0.230), and EIF4A3 (HR = 1.656, 95% CI: 1.289-2.126, *P* < 0.001) were associated with the DFS (Supplementary Table 1). Although in the multivariate Cox regression the SNRPD3 has no significance, it may attribute to the similar expression with the SNRPD2. To prevent missing the information of the SNRPD3, the SNRPD3 was included in the risk score model. The risk score of the hub genes was calculated using the formulae risk score = (0.48) × SNRPD2 expression + 0.16 × SNRPD3 + 0.52 × EIF4A3 expression, based on the Cox regression analysis. The patients were subsequently divided into the high- and low-risk groups (Figure 6(a)). Notably, patients in the low-risk group had an improved DFS and OS than those in the high-risk group (both log-rank *P* < 0.01, Figures 6(b) and 6(c)). The risk score can also predict the metastasis (AUC = 0.70, *P* < 0.01, Figure 7(d)) and disease recurrence (AUC = 0.87, *P* < 0.01, Figure 7(e)) in MM patients. As shown in Figure 7(f), the result demonstrated that the risk score was higher in the metastasis group compared with the nonmetastasis group (2.172 ± 0.327 vs. 1.322 ± 0.1047, *P* < 0.01). Similar result was also been found in the recurrence group compared with the disease-free group (1.966 ± 0.143 vs. 0.6503 ± 0.05832, *P* < 0.01). Time-dependent AUC curves showed that the risk score model had the best powerful ability to predict the MM patient prognosis (Figures 7(g) and 7(h)).

### 3.6. The Association between Risk Score and Clinical Characteristics in MM Patients

The 147 MM patients according to the risk score were divided into the low- (*n* = 78) and the high-risk score groups (*n* = 69). Notably, there were no significant differences in gender, age, American Society of Anaesthesiology (ASA) grade, tumor location, anemia, hypoproteinemia, NLR, MLR, PLR, and SII (Table 1). The high-risk group had a significantly higher pathology T stage (*P* < 0.001), pathology N stage (*P* < 0.001), and pathology M stage (*P* = 0.018) than the low-risk group.

**Figure 8:**
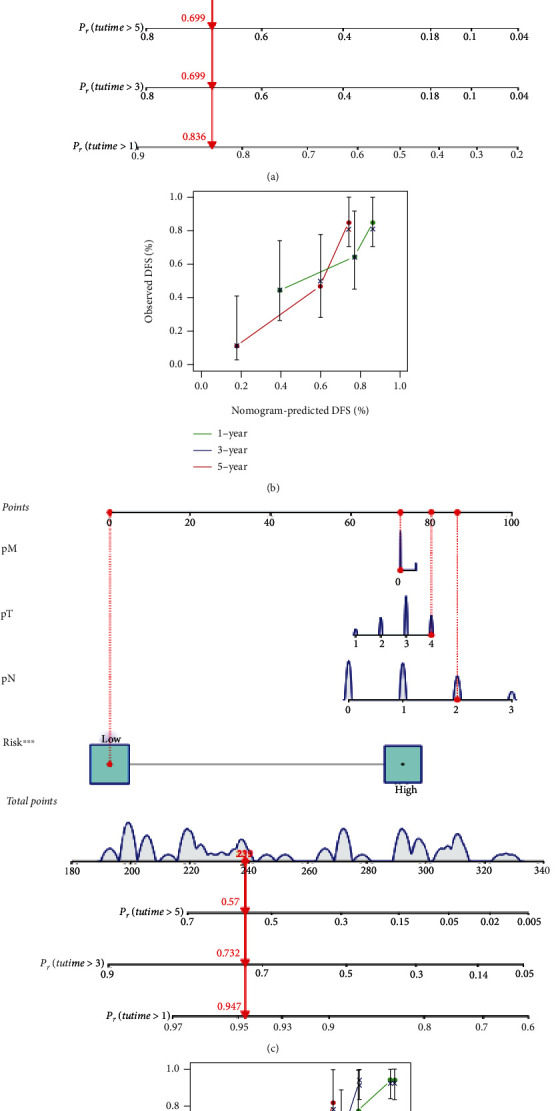
The nomogram model of the prognosis in MM patients. Nomogram predicting DFS (a) and calibration curves in DFS (b). Nomogram predicting OS (c) and calibration curves in OS (d).

The Cox regression analysis was subsequently performed to determine the prognostic factors in MM patients. The univariate Cox regression analysis revealed that higher pathological T stage (HR = 2.129, *P* < 0.001), pathological N stage (HR = 2.613, *P* < 0.001), and a higher risk score (HR = 1.533, *P* < 0.001) were independently associated with the DFS in MM patients. A higher pathological N stage (HR = 2.034, *P* = 0.006) and higher risk score (HR = 1.338, *P* < 0.001) remained significantly associated with the DFS in MM patients in the multivariate Cox regression analysis (Table 2). Moreover, based on the results from multivariate Cox regression, a predicting nomogram for DFS was developed, as shown in Figures 8(a) and 8(b). By summing up the score of each variable, a straight line could be drawn to obtain the predicted the DFS rate.

The Cox univariate analysis further revealed that a higher pathological T stage (HR = 2.030, *P* < 0.001), pathological N stage (HR = 2.459, *P* < 0.001), pathological M stage (HR = 3.703, *P* < 0.001), and a higher risk score (HR = 1.539, *P* < 0.001) were independently associated with the OS in MM patients. A higher pathological N stage (HR = 1.899, *P* < 0.001), pathological M stage (HR = 2.486, *P* = 0.002), and higher risk score (HR = 1.400, *P* < 0.001) remained significantly associated with increased risk of OS in MM patients in the multivariate Cox regression analysis (Table 3). Moreover, based on the results from the multivariate Cox regression, a predicting nomogram for OS was developed, as shown in Figures 8(c) and 8(d). By summing up the score of each variable, a straight line could be drawn to obtain the predicted the OS rate.

### 3.7. Validation of the Risk Score in the External Data Set

**Figure 9:**
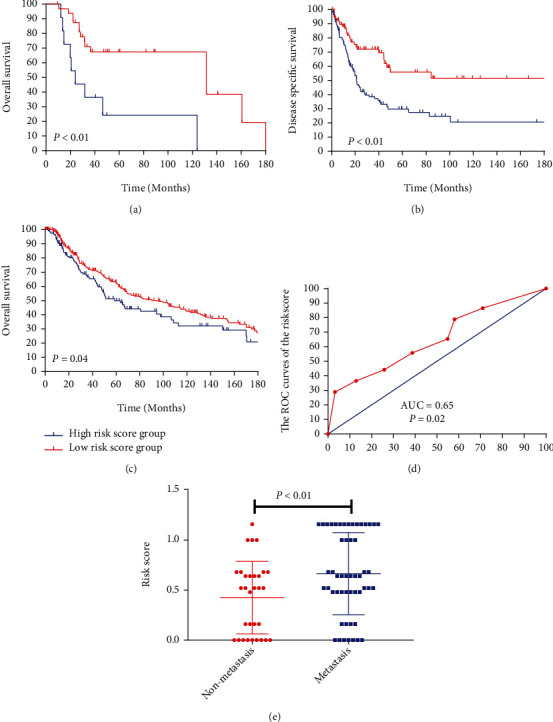
Validation of the risk score model in the external data set. (a–c) The K-M analysis of the risk score model in the external data set, GSE19234 (a), GES65904 (b), and TCGA (c) (all *P* < 0.05). (d) ROC curves and AUC statistics to evaluate the predictive efficiency of the risk score model to distinguish diseases free from metastasis in melanoma patients in GSE8401 data set (AUC = 0.65, *P* = 0.02). (e) The risk score in the melanoma patients (metastasis group vs. nonmetastasis group: 0.66 ± 0.057 vs. 0.24 ± 0.09, *P* < 0.01).

To further explore the risk score model can predict the prognosis and metastasis in MM patients. Based on the X-tile result, we calculated the risk score in each external data set. Then, the patients were divided into two groups, high-risk score group and low-risk score group. The K-M analysis demonstrated that the risk score can distinguish the prognosis in the each external data set (GSE19234, *P* < 0.01, Figure 9(a); GSE65904, *P* < 0.01, Figure 9(b); TCGA, *P* = 0.04, Figure 9(c)). In the GSE8401 data set, the metastasis group had higher risk score compared with the nonmetastasis group (*P* < 0.01, Figure 9(f)). And the ROC curve also demonstrated that risk score model could efficiently predict the metastasis in GSE8401 data set (*P* = 0.02, AUC = 0.65, Figure 9(e)).

### 3.8. GSEA Analysis and Correlation Analysis

**Figure 10:**
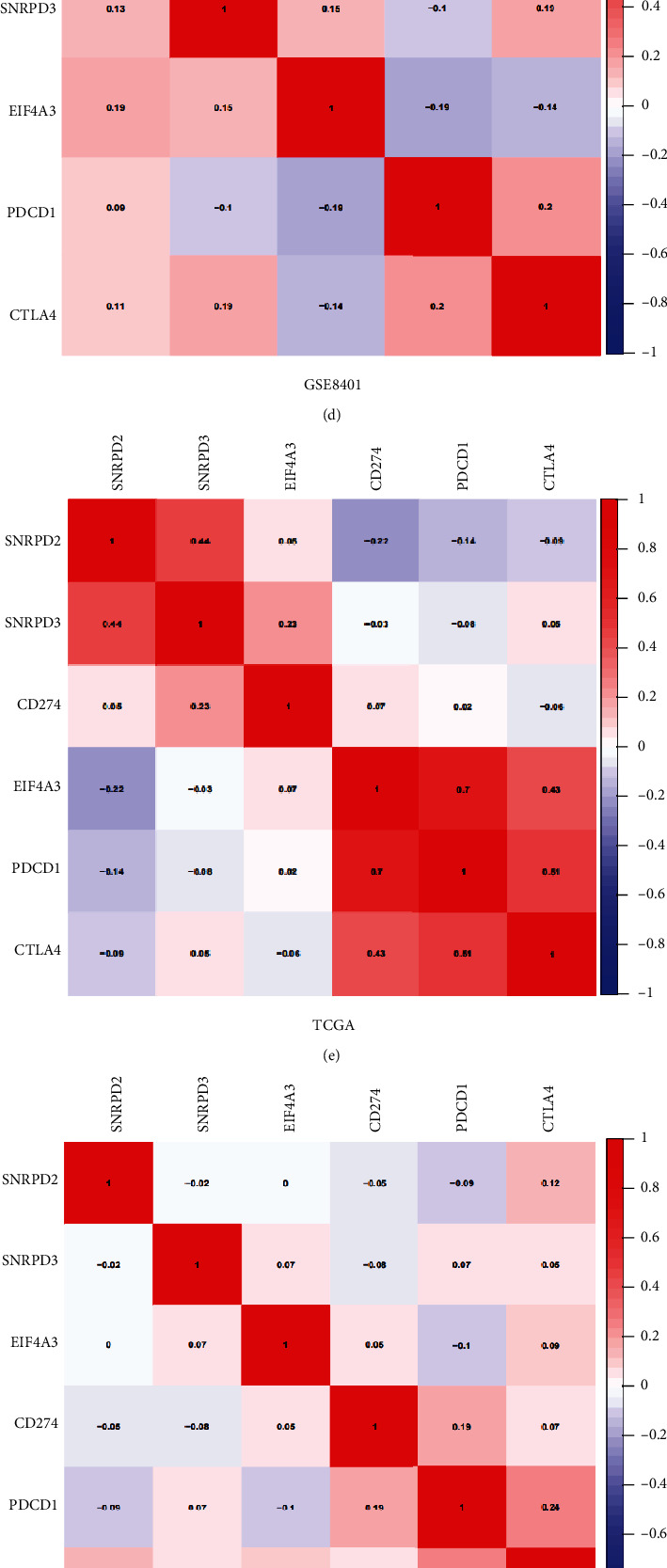
GSEA using the GSE8401 data set and the Pearson analysis of the hub genes. (a) The associated KEGG pathways of the SNRPD2. (b) The associated KEGG pathways of the SNRPD3. (c) The associated KEGG pathways of the EIF4A3. Pearson analysis was performed to analyze the PD1, PD-L1, CTLA4, and hub genes in GSE8401 (d), TCGA (e), GSE65904 (f), and GSE19234 (g). TIMER analysis of the relationship between hub genes, SNRPD2 (h), SNRPD3 (i), and EIF4A3 (j) and tumor-infiltrating immune cells.

GSEA was conducted to determine the potential mechanism for hub gene involvement in metastasis in MM patients. As shown in Figures 10(a)–10(c), the three hub genes were all involved in the immunodeficiency. To further explore the relationship between hub genes and immunotherapy sites, the Pearson analysis was performed to analyze the PD1, PD-L1, and CTLA4 genes. The result demonstrated that the SNRPD2 was associated with the PD-L1in the TCGA data set (*r* = −0.27, *P* < 0.01, Figure 10(e)). However, we could not find the similar result in the other data set (Figures 10(d), 10(f), and 10(g)). Moreover, the TIMER database was employed to analyze the hub gene association with the tumor-infiltrating immune cells. The result demonstrated that the SNRPD2 was associated with the tumor-infiltrating immune cells (all *P* < 0.05, Figure 10(h)), and EIF4A3 was associated with the B cells, CD8+ T cells, neutrophil, and dendritic cells (*P* < 0.05, Figure 10(j)). However, the SNRPD3 was uncorrelated with the tumor-infiltrating immune cells (all *P* > 0.05, Figure 10(i)).

## 4. Discussions

Metastasis is a tough problem in the treatment of cancer patients. In the MM patients with metastasis, the 5-year survival rate only remains 20%-30% [5]. Thus, to explore the efficiency biomarkers that could precisely predict the patients who have distant metastasis is important. In the present study, based on the WGCNA, we identified the three hub genes, SNRPD2, SNRPD3, and EIF4A3. Then, we identified that the hub genes were associated with the metastasis and prognosis in MM patients in the internal and external data sets. Finally, a risk score model was constructed to distinguish the metastasis and prognosis in the MM patients.

The early/local MM could be curable by the adequate surgery, and the 5-year survival rate for patients with early/local MM is over 90% [28]. However, most MM patients had metastasis at the time of diagnosis. In our data, for the patients diagnosed with MM, the rate of the pathology stage II/III was 72.8% (107/147). Thus, to explore the efficiency biomarkers that could precisely predict the patients who have distant metastasis is important. However, to screen the hub genes basing on the traditional differential expressed genes would miss important information. WGCNA is an effective method to discover the relationship between individual genes and the clinical characteristics in cancer [10–12]. Thus, the WGCNA was performed to screen the hub genes associated with the metastasis in the MM from the GSE8401 which contained the metastatic and nonmetastatic MM patients. The result from the WGCNA revealed that the green module is the hub module. And the GO and KEGG analysis demonstrated that the green module was associated with the MAPK signaling pathway and cell cycle signaling pathway which has already been reported associated with the metastasis in the MM [29, 30]. Then, based on the PPI and MCODE analysis, the three hub genes, SNRPD2, SNRPD3, and EIF4A3, had been screened out and been identified as the useful biomarkers to predict the metastasis in the MM patients by ROC curve and expression value analysis in the GSE8401.

The MM could occur in the skin and other mucous. In Europe and the US, the MM often triggered by ultraviolet and occurred in the skin [1]. In China, the MM occurred in the mucous except the skin nearly 50% (51.0%, 75/147, in our data) [31]. Several studies have been reported the useful biomarkers to predict the metastasis in the MM. Wu et al. [32] described that the EIF3B acted as the oncogene in the melanoma and affected the progression and immunotherapy resistance development in European and US patients. Meng et al. [33] introduced that increased PRRX1 expression is independently a prognostic factor of poorer OS and metastasis-free survival in patients with uveal melanoma in US patients. A single-cell analysis was performed and constructed in a prognostic model in European and US patients [34]. However, the biomarkers were not identified in the Asian. Whether the biomarkers could be used in the mucous MM or Chinese is still unknown. To explore effective biomarkers that can predict the metastasis in the skin MM and mucous MM is important. In the present study, we searched the GEO database and found the different source MM data to identify the hub genes which had strong ability to predict the metastasis and prognosis in the skin MM and mucous MM. However, the four external data sets which we enrolled in the present study were all came from Europe and the US, and Chinese data set is lacking. To further identify the hub genes could predict the metastasis and prognosis in the Chinese MM. The IHC analysis was performed to detect the hub gene expression in the Chinese MM patients. The results were consistent with the Europe and US result. The above result revealed that the hub genes had a strong ability to predict the metastasis and prognosis in the multiracial MM patients and different site MM.

The three hub genes, SNRPD2, SNRPD3, and EIF4A3, had been reported in the several studies. SNRPD2 and SNRPD3 belong to the small nuclear ribonucleoprotein Sm family, which participate in the RNA splicing reaction [35]. Accumulating evidence indicates that Sm core proteins influence the profile of alternative splicing [36, 37]. There are several studies reporting that the SNRPD3 was the important composition of the spliceosome [38]. The depletion of the SNRPD3 would cause lethal defects in key steps of mitosi [39, 40]. EIF4A3 is a member of the DEAD-box protein family [41–43], and it is also a core component of the splicing-dependent multiprotein exon junction complex [44]. However, there have been few study reporting the SNRPD2 and SNRPD3 functions in the cancer. In the present study, we found that the SNRPD2 and SNRPD3 acted as the oncogenes in the MM patients, and high expressions of the SNRPD2 and SNRPD3 were associated with the metastasis and worse prognosis. In the present study, we found that the SNRPD2 was associated with the immunodeficiency function, PD-L1 expression in the TCGA data set, and tumor-infiltrating immune cells in the TIMER. The above result suggests that the SNRPD2 may affect the immune response to regulate prognosis and metastasis in MM patients. Previous studies have demonstrated that EIF4A3 is also highly expressed in many tumors, such as lung cancer [45], breast cancer [46], pancreatic cancer [47], colorectal cancer [48], and MM [49]. The result was similar to the present study. We identified that the high expression of the EIF4A3 was associated with the metastasis and worse prognosis in the several data sets. Moreover, the function of the EIF4A3 was explored in the present study, and the result revealed that the EIF4A3 associated with the several tumor-infiltrating immune cells, including B cells, CD8+ T cells, neutrophil, and dendritic cells. The result indicated that the hub genes may affect the immune response to regulate the prognosis and metastasis in the MM patients.

The risk factor model and nomogram model have been reported in several cancers, such as MM, lung, and rectal cancers [27, 33, 50]. In the present study, based on the IHC result, the risk factor model was constructed and was identified as the effective model to predict the metastasis and prognosis in Chinese MM patients. Additionally, the risk model was verified in the external data sets which came from Europe and the US. Moreover, the time-dependent ROC curve further demonstrated that the model had the best AUC value than the single hub gene in predicting DFS and OS in MM patients. Notably, the risk score model also had powerful ability to predict the metastasis and prognosis in external data sets. Succinctly, the risk factor model and nomogram had a strong predictive ability in predicting the prognosis in MM patients.

Despite the notable findings of this study, it was limited by several factors. The WGCNA algorithm was based on the external data set from Europe and it may be different with the Chinese patients. Secondly, the hub genes were identified by the external microarray profiling and IHC analysis. It should be verified by the more data sets. Thirdly, the function of the hub genes was explored by the bioinformatics methods without any validation assays to verify their correctness. Future studies should thus include larger sample sizes and conduct validation assays using in vitro and in vivo experiments to enhance their comprehensiveness.

## 5. Conclusion

In conclusion, through the WGCNA, we screened the three hub genes, SNRPD2, SNRPD3, and EIF4A3. Then, the three hub genes were identified and validated as effective predictors for metastasis and prognostic factor in MM patients in external and internal data sets from Europe, the US, and China. And the SNRPD2 had the powerful predictive ability in the MM. The risk factor model was constructed and verified as a powerful model to predict metastasis and prognosis in MM patients. Nevertheless, more insightful molecular mechanisms are warranted in future studies.

## Figures and Tables

**Table 1:** Baseline characteristics in melanoma patients stratified by risk group.

Characteristics	Low-risk group (*n* = 78)	High-risk group (*n* = 69)	*P* value
Sex (%)			0.096
Male	49 (62.8)	33 (47.8)	
Female	29 (37.2)	36 (52.2)	
Age (years)	58.38 ± 15.04	58.06 ± 13.54	0.891
ASA score (%)			0.781
1	46 (59.0)	44 (63.8)	
2	28 (35.9)	21 (30.4)	
3	4 (5.1)	4 (5.8)	
Tumor location			0.137
Skin	43 (55.1)	29 (42.0)	
Other mucosa	35 (44.9)	40 (58.0)	
Pathological T stage (%)			**<0.001**
1	5 (6.4)	3 (4.3)	
2	30 (38.5)	6 (8.7)	
3	28 (35.9)	39 (56.5)	
4	15 (19.2)	21 (30.4)	
Pathological N stage (%)			**<0.001**
N0	43 (55.1)	16 (23.2)	
N1	25 (32.1)	26 (37.1)	
N2	9 (11.5)	22 (31.9)	
N3	1 (1.3)	5 (7.2)	
Pathological M stage (%)			**0.018**
M0	74 (94.9)	56 (81.2)	
M1	42 (5.1)	13 (18.8)	
Anemia	8 (10.3)	5 (7.2)	0.573
Hypoproteinemia	2 (2.6)	2 (2.9)	1.000
NLR	1.52 ± 0.19	1.54 ± 0.18	0.496
MLR	0.46 ± 0.20	0.0.47 ± 0.21	0.584
PLR	238.74 ± 89.42	252.09 ± 102.77	0.401
SII	344.27 ± 99.50	339.41 ± 79.15	0.746

ASA: American Society of Anesthesiologists; NLR: neutrophil-to-lymphocyte ratio; SII: systemic immune-inflammation index; MLR: monocyte-to-lymphocyte ratio; PLR: platelet-to-lymphocyte ratio.

**Table 2:** Cox regression analysis of predictive factors for disease-free survival in melanoma patients (*n* = 147).

Variables	Univariate analysis			Multivariate analysis		
	HR	95% CI	*P* value	HR	95% CI	*P* value
Sex, male/female	0.992	0.648-1.519	0.971			
Age	0.990	0.976-1.004	0.174			
ASA	1.071	0.750-1.528	0.707			
Pathological T stage	2.129	1.605-2.824	**<0.001**	1.201	0.833-1.731	0.327
Pathological N stage	2.613	2.040-3.347	**<0.001**	2.034	1.433-2.887	**<0.001**
Tumor location, skin/other mucosa	1.306	0.852-2.001	0.221			
Risk score	1.533	1.348-1.743	**<0.001**	1.338	1.156-1.549	**<0.001**
Anemia	1.203	0.581-2.491	0.679			
Hypoproteinemia	0.909	0.224-3.696	0.894			
NLR	1.025	0.323-3.252	0.966			
MLR	1.587	0.591-4.259	0.359			
PLR	1.001	0.999-1.003	0.428			
SII	1.000	0.998-1.002	0.922			

HR: hazard ratio; CI: confidence interval; ASA: American Society of Anesthesiologists; NLR: neutrophil-to-lymphocyte ratio; SII: systemic immune-inflammation index; MLR: monocyte-to-lymphocyte ratio; PLR: platelet-to-lymphocyte ratio.

**Table 3:** Cox regression analysis of predictive factors for overall survival in melanoma patients (*n* = 147).

Variables	Univariate analysis			Multivariate analysis		
	HR	95% CI	*P* value	HR	95% CI	*P* value
Sex, male/female	0.821	0.695-1.582	0.821			
Age	1.009	0.995-1.024	0.209			
ASA	1.356	0.964-1.905	0.080			
Pathological T stage	2.030	1.547-2.664	**<0.001**	1.208	0.844-1.729	0.303
Pathological N stage	2.459	1.927-3.137	**<0.001**	1.899	1.352-2.667	**<0.001**
Pathological M stage	3.703	2.116-6.482	**<0.001**	2.486	1.407-4.392	**0.002**
Tumor location, skin/other mucosa	1.243	0.825-1.876	0.299			
Risk score	1.539	1.356-1.746	**<0.001**	1.400	1.224-1.603	**<0.001**
Anemia	0.902	0.437-1.865	0.782			
Hypoproteinemia	0.864	0.213-3.511	0.838			
NLR	2.268	0.756-6.808	0.144			
MLR	1.054	0.374-2.969	0.920			
PLR	1.000	0.997-1.002	0.4682			
SII	0.999	0.997-1.001	0.336			

HR: hazard ratio; CI: confidence interval; ASA: American Society of Anesthesiologists; NLR: neutrophil-to-lymphocyte ratio; SII: systemic immune-inflammation index; MLR: monocyte-to-lymphocyte ratio; PLR: platelet-to-lymphocyte ratio.

## Data Availability

The data generated or analyzed during this study are available from the corresponding author upon reasonable request.

## Abbreviations

WGCNA: Weighted gene coexpression network analysis
GEO: Gene Expression Omnibus
TCGA: The Cancer Genome Atlas
STRING: Search Tool for the Retrieval of Interacting Genes
PPI: Protein-protein interaction
GSEA: Gene set enrichment analysis
ROC: Receiver operating characteristic curve
AUC: Area under the curve.

## Supplementary Materials

Supplementary Materials Supply Table 1: Cox regression analysis of three hub genes for disease-free survival in melanoma patients (*n* = 147).
