# Supplementary material for: Identification of Three Genes Associated with Metastasis in Melanoma and Construction of a Predictive Model: A Multiracial Identification
Source: J Oncol. 2022 May 21;2022:4567063. doi: 10.1155/2022/4567063 (PMC9148232; doi:10.1155/2022/4567063)
Supplement: Supplementary Materials — Supply Table 1: Cox regression analysis of three hub genes for disease-free survival in melanoma patients (n = 147). [file 4567063.f1.pdf]

Supply TABLE 1 Cox regression analysis of three hub genes for disease-free survival in melanoma patients (n=147)

Variables	Multivariate analysis		
	**HR**	**95% CI**	***P* value**
SNRPD2	1.603	1.236-2.079	**<0.001**
SNRPD3	1.174	0.904-1.525	0.230
EIF4A3	1.656	1.289-2.126	**<0.001**

HR, hazard ratio; CI, confidential interval
